# Comparative Transcriptome and Proteome Analysis of Salt-Tolerant and Salt-Sensitive Sweet Potato and Overexpression of *IbNAC7* Confers Salt Tolerance in *Arabidopsis*

**DOI:** 10.3389/fpls.2020.572540

**Published:** 2020-08-27

**Authors:** Xiaoqing Meng, Siyuan Liu, Tingting Dong, Tao Xu, Daifu Ma, Shenyuan Pan, Zongyun Li, Mingku Zhu

**Affiliations:** ^1^Institute of Integrative Plant Biology, School of Life Sciences, Jiangsu Normal University, Xuzhou, China; ^2^Jiangsu Key Laboratory of Phylogenomics & Comparative Genomics, School of Life Science, Jiangsu Normal University, Xuzhou, China; ^3^Jiangsu Xuzhou Sweet Potato Research Center, Chinese Academy of Agricultural Sciences (CAAS), Xuzhou, China

**Keywords:** differentially expressed genes, IbNAC7, salt stress, sweet potato, transcriptome and proteome analysis

## Abstract

Salt stress is one of the major devastating factors affecting the growth and yield of almost all crops, including the crucial staple food crop sweet potato. To understand their molecular responses to salt stress, comparative transcriptome and proteome analysis of salt-tolerant cultivar Xushu 22 and salt-sensitive cultivar Xushu 32 were investigated. The results showed the two genotypes had distinct differences at the transcription level and translation level even without salt stress, while inconsistent expression between the transcriptome and proteome data was observed. A total of 16,396 differentially expressed genes (DEGs) and 727 differentially expressed proteins (DEPs) were identified. Wherein, 1,764 DEGs and 93 DEPs were specifically expressed in the tolerant genotype. Furthermore, the results revealed that the significantly upregulated genes were mainly related to the regulation of ion accumulation, stress signaling, transcriptional regulation, redox reactions, plant hormone signal transduction, and secondary metabolite accumulation, which may be involved in the response of sweet potato to salt stress and/or may determine the salt tolerance difference between the two genotypes. In addition, 1,618 differentially expressed regulatory genes were identified, including bZIP, bHLH, ERF, MYB, NAC, and WRKY. Strikingly, transgenic *Arabidopsis* overexpressing *IbNAC7* displayed enhanced salt tolerance compared to WT plants, and higher catalase (CAT) activity, chlorophyll and proline contents, and lower malondialdehyde (MDA) content and reactive oxygen species (ROS) accumulation were detected in transgenic plants compared with that of WT under salt stress. Furthermore, RNA-seq and qRT-PCR analysis displayed that the expression of many stress-related genes was upregulated in transgenic plants. Collectively, these findings provide revealing insights into sweet potato molecular response to salt stress and underlie the complex salt tolerance mechanisms between genotypes, and *IbNAC7* was shown as a promising candidate gene to enhance salt tolerance of sweet potato.

## Introduction

Plants are often permanently exposed to a variety of abiotic stresses, such as salinity, drought, heat, and cold. Among them, salt stress is the major devastating factor affecting crop growth and productivity (Parida and Das, 2005). About 9 billion hm² of land worldwide is affected by salt stress, accounting for at least 6% of the total land area, and 50% of irrigated land is facing salinity problems (Flowers, 2004; Acosta-Motos et al., 2015; Kumar et al., 2017). Therefore, salt stress is a widespread and common feature of massive lands, and plants have evolved various mechanisms to tolerate salt stress. Presently, a great deal of documents have identified and characterized the components of the salt stress signaling network. For instance, the first established plant abiotic stress signaling pathway, the SOS pathway, is a calcium-dependent protein kinase pathway that plants use for salt stress signaling and Na^+^ resistance (Zhu, 2002; Zhu, 2016). However, the specific physiological and molecular mechanisms of salt tolerance remain largely unknown in plants.

Plants’ response to salt stress involves sophisticated and diverse tolerance mechanisms that are activated and integrated by the transcription of thousands of genes with enormous biological roles (Kant et al., 2007; Zhu, 2016). Transcription factors (TFs) are pivotal because they are involved in the regulation of signal transduction and the transcription of many stress-related genes, such as bZIP, MYB, WRKY, AP2/ERF, and NAC proteins (Erpen et al., 2018; Zhu et al., 2018; Li et al., 2019; Yang et al., 2019). NACs are one of the largest plant-specific TFs, and have been widely isolated from various species, such as *Arabidopsis* and rice (Nuruzzaman et al., 2010), potato (Singh et al., 2013), tomato (Jin et al., 2020) and maize (Wang et al., 2020). Typically, NACs have an N-terminal NAC domain consisting of approximately 150 conserved amino acids involved in DNA or protein binding, and the NAC domain can be divided into five sub-domains (A–E). Studies have shown that the diverse C-terminal is considered as a transcription regulatory region that can activate or repress gene expression (Puranik et al., 2012; Mohanta et al., 2020).

Significant progress has critically demonstrated that numerous NACs in various plant species are involved in diverse biological processes, especially in response to biotic and abiotic stress, such as drought, salt, and cold (Puranik et al., 2012; Mathew and Agarwal, 2018). A large number of documents showed that transgenic plants overexpressing a NAC gene have enhanced stress tolerance, illustrating that NACs are promising candidate factors for genetic engineering of crops under adverse conditions (Tran et al., 2010; Marques et al., 2017). For example, the over accumulation of *Arabidopsis ANAC019*, *ANAC055*, and *ANAC072* resulted in improved drought tolerance and modulated the transcription of numerous stress- and ABA-related genes (Tran et al., 2004). Transgenic rice that overexpressed *SNAC1* or *SNAC2* genes displayed obviously improved tolerance to drought and salt, and the expression of lots of stress-related genes was upregulated in *SNAC2*-overexpressing plants (Hu et al., 2006; Nakashima et al., 2007). Presently, Hou et al. (2020) find that overexpression of *CaNAC064* confers cold tolerance, while down-regulation of *CaNAC064* displays the opposite performance in transgenic pepper (Hou et al., 2020). Furthermore, plenty of studies have suggested that NACs respond to various abiotic stresses *via* the downstream actions of hormones including ABA and ethylene. For instance, overexpression of multiple NACs results in altered ABA sensitivity in transgenic plants (Marques et al., 2017; He et al., 2019). And multiple NAC TFs function directly to the ABA biosynthesis-related genes, such as both *Arabidopsis* ATAF1 and rice OsNAC2 can directly bind to the promoter of *NCED3* gene (Jensen et al., 2013; Mao et al., 2017).

Sweet potato (*Ipomoea batatas* L.) is the only crop with starch storage roots in the Convolvulaceae family (Liu, 2017; Arisha et al., 2020), and is one of the most important food crops, ranking seventh in the world and fourth in China (Meng et al., 2018). Sweet potato is widely applied for human food, animal feed, and for manufacturing starch and alcohol. Because of its ability to adapt various agro-ecological conditions, sweet potato has ensured food supply and safety in many developing countries, but its yield is still reduced by many biotic and abiotic stresses (Liu, 2017). Presently, many stress-related genes have been identified in sweet potato. For example, IbABF4 TF confers drought and salt tolerance in transgenic sweet potato and *Arabidopsis* (Wang W. et al., 2019), and IbMYB116 TF improves drought tolerance in transgenic *Arabidopsis* (Zhou et al., 2019). Our previous study also displayed that overexpression of a AP2/ERF gene, *IbCBF3*, increased the cold and drought tolerance in transgenic sweet potato (Jin et al., 2017). However, the roles of most stress-responsive genes in sweet potato remain largely unknown. At the same time, although extensive reports have largely revealed the importance of transcriptional regulations under salt stress, few studies have studied the regulation of translation level. In this study, transcriptome and proteome analysis were simultaneously performed in two contrasting sweet potato cultivars Xushu 22 (salt-tolerant, abbreviated as Xu22) and Xushu 32 (salt-sensitive, abbreviated as Xu32) we previously identified (Yu et al., 2016) under control and salt-exposed conditions. We found that overexpression of *IbNAC7* obviously enhanced the salt tolerance of transgenic *Arabidopsis*. This study identified crucial genes/proteins and pathways between the two contrasting cultivars under salt stress, and provided fundamental insights into the molecular mechanisms underlying sweet potato stress tolerance.

## Materials and Methods

### Plant Materials and Cultural Conditions

The tuberous roots of two contrasting sweet potato cultivars Xu22 and Xu32 with different salt tolerance (Yu et al., 2016) were placed in the greenhouse, and then the shoots with functional leaves were cut and hydroponic culture in 1/4 Hoagland solutions in a plant growth chamber timed for 16 h days (25°C) and 8 h nights (20°C). Hoagland solution was replaced every three days, afterwards uniform seedlings of both cultivars with five to six functional leaves and 8 to 10 cm fibrous roots were exposed to 150 mM NaCl for 24 h, and fibrous roots before and after salt stress were harvested for transcriptome analysis. All the samples were immediately immersed in liquid nitrogen and stored at −70°C.

### Transcriptome Analysis

The fibrous roots of Xu22 and Xu32 were harvested from six different plants for transcriptome analysis of each set with three biological replicates. 1 μg RNA from each sample was employed as input material, and 12 samples were transferred to Biomarker technology Co. Ltd. (Beijing, China) for transcriptome sequencing and assembly. Clean reads were achieved by removing reads containing adapters, poly-N and low-quality reads from raw reads, and then the filtered sequences were used for downstream analysis. Hisat2 was used to map with the sweet potato reference genome (https://ipomoea-genome.org/). Gene expression was estimated by read counts, and genes with adjusted P-values (false discovery rate, FDR) < 0.05 detected by DESeq (Anders and Huber, 2010) and |log2 (fold change)| >1 were considered as DEGs. GO enrichment analysis was implemented by GOseq (Young et al., 2010), and the statistical enrichment of KEGG pathways were tested by KOBAS (Mao et al., 2005).

### Proteome Analysis

The iTRAQ analysis of the proteome was carried out by Biomarker technology Co. Ltd. (Beijing, China) as described in our previous report (Dong et al., 2019). Briefly, total proteins were extracted from 12 samples, and their purity was detected by 10% SDS-PAGE. The peptides were dried by vacuum centrifugation after trypsin digestion, and then labeled using iTRAQ^®^ Reagent-8PLEX Multiplex Kit (Sigma). About 600 μg of the labeled peptide mix was fractionated by a C18 column on Rigol L3000 HPLC. The obtained spectra were searched against the sweet potato reference genome using the PD 2.2 (Thermo). The protein quantitation was estimated by the Mann-Whitney test, and fold change >1.2 was applied to screen the differential proteins. GO and InterPro (IPR) analysis were carried out by the InterProScan-5 against multiple protein databases, such as Pfam, SMART and ProSiteProfiles, and KEGG database was employed to analyze the protein pathways.

### Construction of Overexpression Vector and *Arabidopsis* Transformation

The coding region of *IbNAC7* was inserted into the pBI121 binary vector driven by the CaMV 35S promoter using the primers *Ov-IbNAC7*-F/R (**Supplementary Table S1**). The vector was then transferred into *A. tumefaciens* GV3101, and transgenic *Arabidopsis thaliana* (Columbia-0) were produced and further to obtain homozygous T3 seeds according to the methods described by Zhang et al. (2006). The expression of *IbNAC7* in transgenic plants was confirmed by qRT-PCR using the CFX96™ Real-Time System (Bio-Rad, USA) as described in our previous report (Meng et al., 2019). The relative expression was normalized to *EF1α* (**Supplementary Table S1**), and the transcription was further calibrated using the transgenic line with the lowest *IbNAC7* expression.

### Assays for NaCl Stress Tolerance of Transgenic *Arabidopsis*

For germination greening rate assay, seeds of WT and T3 transgenic lines were sterilized with 1% NaClO for 10 min, and then sown on 1/2 MS medium with 0 (as a control) and NaCl (100 and 150 mM). About 180 seeds per line were used in each assay, and the germination greening rate was calculated after 10 d. For root length assay, 4-d-old seedlings of WT and transgenic lines were selected based on the consistency of root length, and were vertically cultured on 1/2 MS medium with 0 (as a control) and 120 mM NaCl in a growth chamber. The length of the primary roots was measured after 10 d. For salt stress in soil, 7 d-old seedlings of WT and transgenic lines obtained on 1/2 MS medium were transferred to pots in a greenhouse; two weeks later, plants were randomly selected for salt tolerance assay. One group was watered normally as a control, and the other group was irrigated with 200 mM NaCl solution from the bottom of pots every 3 d. Pictures were taken 15 d later to record the phenotype.

### RNA-Seq Analysis and Salt Tolerance Evaluation of Transgenic *Arabidopsis*

The 10-d-old seedlings of WT and transgenic line 8, which were vertically cultured on 1/2 MS medium, were soaked in liquid 1/2 MS medium containing 100 mM NaCl solution for 6 h, and then the whole plants before and after NaCl treatment were collected and used for RNA-seq analysis as described above, except that clean reads were mapped to the *Arabidopsis* reference genome (https://www.arabidopsis.org/).

In addition, the leaves of WT and transgenic plants in the soil under normal and salt stress for 15 d were used to determine stress-related physiological indicators. Chlorophyll, proline and malondialdehyde (MDA) contents, and catalase (CAT) activity were detected using corresponding test kits (for plant) purchased from Nanjing Jiancheng Bioengineering Institute (Nanjing, China) according to the manufacturer’s protocols. Superoxide radicals were histochemically detected using the nitroblue tetrazolium (NBT) and dead cells were visualized by trypan blue (TB) staining according to the procedures described by Lee et al. (2012).

### RNA Extraction and qRT-PCR Analysis

Total RNA was isolated from all samples using an RNA extraction kit (TianGen, Beijing, China) according to the manufacturer’s instructions. 2 μg RNA was reverse-transcribed using PrimeScript reverse transcriptase with gDNA Eraser (TaKaRa, Dalian, China) using the mix of Oligo dT Primer and Random 6 mers. qRT-PCR experiments were carried out on a CFX96™ Real-Time System (Bio-Rad, USA) as described in our previous report (Meng et al., 2019). The *Arabidopsis EF1α* gene was selected as the internal standard. All qRT-PCR primers are listed in **Supplementary Table S1**, and each gene was performed with three independent biological replicates, and three technical replicates for each biological replicate.

### Statistical Analyses

The data were analyzed by one-way analysis of variance (ANOVA) and means different were significant by a Dunnett’s test at P < 0.05. Statistical analyses were conducted with SPSS software 20 version (IBM Corp., USA).

## Results

### Enrichment Analysis of the DEGs (Differentially Expressed Genes) Between Xu22 and Xu32 Under Salt Stress

Our previous studies have shown that better ion homeostasis and nitrogen metabolism make Xu22 more salt tolerant than Xu32 (Yu et al., 2016). To further understand the molecular mechanism underlying the two contrasting sweet potato cultivars with different salt tolerance, their fibrous roots from control and salt-exposed conditions (total 12 samples) were collected for transcriptome sequencing using the Illumina HiSeq 2500 platform. A total of 40,767,744 to 55,915,888 clean reads were generated, of which 73.73% to 75.21% clean reads were mapped to the sweet potato genome, and less than 3.98% of the reads were mapped to multiple sites (**Supplementary Table S2**). The RNA data in the three biological replicates showed high expression correlation (R^2^ ≥ 0.897) except Xu22-CR2 (R^2^ ≤ 0.738, **Supplementary Figure S1**), thus these assembled sequences except Xu22-CR2 are appropriately employed for downstream analysis.

**Figure 1:**
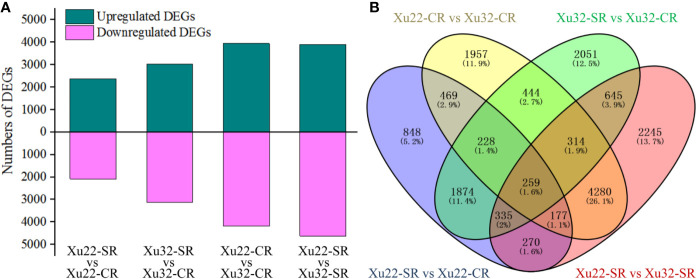
Overview and Venn diagram of upregulated or downregulated genes in fibrous roots of two sweet potato genotypes under normal and salt-treated conditions at a level of |log2 (fold change)| > 1 and FDR < 0.05. **(A)** The total number of differentially expressed genes (DEGs) found in the fibrous roots of Xu22 and Xu32 under control and salt-treated conditions. **(B)** The four-way Venn diagram in Xu22 and Xu32 suggests that the DEGs were genotype-specific and salt-responsive. Overlapping regions indicate co-expressed DEGs among different data sets, and numbers in only one circle represent DEGs expressed in only one library.

The read counts value was calculated to profile the transcription level of genes. A total of 16,396 DEGs were identified from the fibrous root libraries under normal and salt-treated conditions based on the FDR < 0.05, and |log2 (fold change)| > 1 (**Supplementary Figure S2**, **Supplementary Table S3**). Among them, 4,460 and 6,150 DEGs were from Xu22-SR vs Xu22-CR and Xu32-SR vs Xu32-CR, respectively, 8,128 DEGs were from Xu22 vs Xu32 without salt stress, and 8,525 DEGs were from Xu22 vs Xu32 under salt stress. Interestingly, the total number of salt-responsive genes in Xu32 was larger than in Xu22 under salt stress (both upregulated and downregulated genes) (**Figure 1A**). Venn analysis showed that many DEGs identified were salt stress-responsive and/or genotype-specific. Among them, 1,764 and 3,454 DEGs were specifically expressed in Xu22 and Xu32 under salt stress, respectively, and 2,696 DEGs were expressed in both libraries (**Figure 1B**).

Functional annotations showed that a large number of salt-responsive genes identified in the roots of Xu22 and Xu32 under salt stress are involved in the regulation of ion accumulation, stress signaling, redox reactions, plant hormone signal transduction, and accumulation of secondary metabolites (**Supplementary Table S3**). These pathways have been shown to play pivotal roles in the salt tolerance of many plants (Zhang et al., 2019), and thus may represent the core genes related to salt stress response, despite the differential levels of salt tolerance in sweet potato. Especially, compared to samples from salt-sensitive Xu32, we found that many DEGs encode stress-related proteins/factors, such as NAC TF, zinc finger protein, F-box protein, MAPKKK, DEAD-box RNA helicase, plasma membrane ATPase, calmodulin-binding protein, and cytochrome P450 were uniquely expressed in salt-tolerant Xu22. Furthermore, the expression levels of many DEGs related to WRKY TF, ERF TF, PPR protein, F-box protein, zinc finger protein, ABA-induced protein, potassium transporter, methyltransferase, homeobox protein, cytochrome P450 were also significantly upregulated in Xu22 compare to that in Xu32 under salt stress (**Supplementary Table S3**).

**Figure 2:**
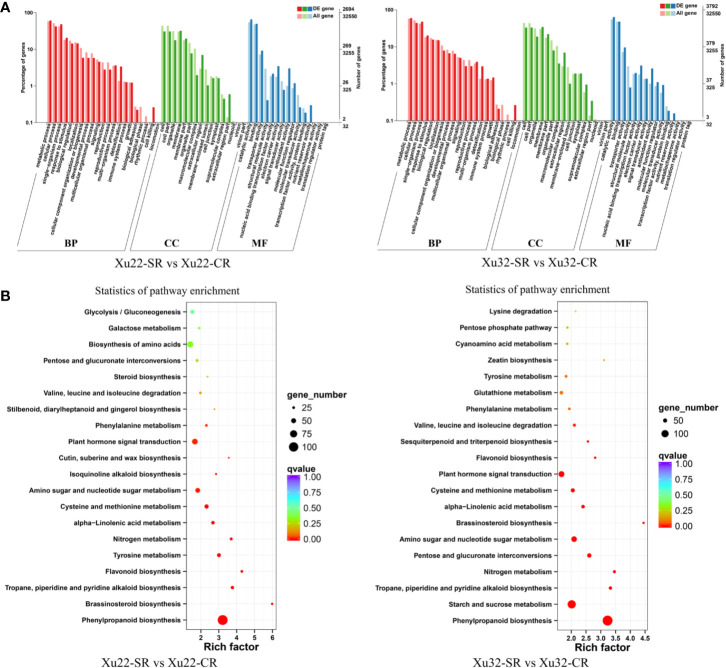
GO classifications and KEGG pathways of the DEGs in the fibrous roots of Xu22 and Xu32 under salt stress. **(A)** GO classifications of the annotated DEGs. The left Y-axis indicates the percentage of DEGs identified, and the right Y-axis indicates the number of DEGs. The DEGs were categorized based on the annotations of GO, and the numbers are displayed according to the biological process (BP), cellular component (CC), and molecular function (MF). **(B)** Enriched KEGG pathways of the DEGs in Xu22 and Xu32 under salt stress. X-axis and Y-axis represent the GeneRatio and the terms of pathways, respectively. Coloring correlates with the q-value. The lower the q-value, the more significant the enrichment. Point size correlates with the numbers of DEGs.

GO annotations of the DEGs showed that over 50 functional terms of the four sets of data were classified (**Supplementary Table S4**). The first seven most enriched functional terms are similar between Xu22 and Xu32 under salt stress. Among them, “catalytic activity” term (ranging from 1,765 to 2,539 DEGs) in molecular function and “metabolic process” term (ranging from 1,640 to 2,731 DEGs) in biological process were the two most common categories in both Xu22 and Xu32 under salt stress (**Figure 2A**, **Supplementary Figure S3A**). The results indicated that the response of sweet potato to salt stress involves intensive metabolic activity and catalytic activity. Strikingly, lots of DEGs were involved in multiple crucial GO terms which are known to be related to plant salt tolerance, such as “antioxidant activity,” “transcription factor activity,” and “response to stimulus.” Moreover, GO analysis also displayed apparent genotype-specific enrichment, suggesting that many important biological process, cellular component and molecular function occurred differently between Xu22 and Xu32 under salt stress (**Supplementary Table S4**).

Besides, KEGG-based DEGs enrichment analysis showed that most KEGG pathways were enriched in both genotypes under salt stress. The most enriched categories are “phenylpropanoid biosynthesis” in both Xu22 (106 DEGs) and Xu32 (143 DEGs) under salt stress, and “ribosome” in Xu22 vs Xu32 under salt stress (137 DEGs) and Xu22 vs Xu32 without salt stress (133 DEGs) (**Figure 2B**, **Supplementary Figure S3B**). Common categories of high enrichment also included “starch and sucrose metabolism,” “carbon metabolism,” “plant hormone signal transduction,” “biosynthesis of amino acids.” However, the “ribosome” pathway in Xu22 was significantly upregulated compared to Xu32 under salt stress (**Supplementary Figure S4**). In particular, several categories were uniquely enriched in Xu22, such as “N-Glycan biosynthesis” and “glucosinolate biosynthesis” (**Supplementary Table S5**). Overall, the data provide valuable knowledge for further understanding the complicated molecular mechanisms of sweet potato responses to salt stress.

### Proteome Analysis and Transcriptome-Proteome Matching Analysis

In many cases, changes in transcription level are not always accompanied by changes in protein abundance, thus comparative proteome analysis was conducted using the same samples as the transcriptome by the iTRAQ. A total of 3,123 proteins were identified in the four sets of data, while the proteome data in the three biological replicates showed a very low expression correlation (each ≤ 0.69) (**Supplementary Figure S5**). Among these proteins, 86.5% (2701), 70.3% (2,196) and 44.7% (1,395) were annotated in the COG, GO, and KEGG databases, respectively (**Supplementary Table S6**). The comparison between different samples showed that a total of 727 DEPs (differentially expressed proteins) were identified, wherein, 124 DEPs (59 upregulated and 66 downregulated) in Xu22, and 262 DEPs (119 upregulated and 143 downregulated) in Xu32 under salt stress were identified. And 93 and 231 DEPs were uniquely expressed in Xu22 and Xu32 under salt stress, respectively. 216 DEPs were from Xu32 vs Xu22 under salt stress, and 125 DEPs were from Xu32 vs Xu22 without salt stress (**Supplementary Figures S6** and **S7**). Commonly upregulated proteins of the two genotypes under salt stress included peroxidase, elongation factor, phosphoglycerate kinase, glycine-rich RNA-binding protein, plasma membrane ATPase, and NADH dehydrogenase. Some DEPs, such as cytochrome P450, cinnamic acid 4-hydroxylase, lipoxygenase, and DNA repair protein, were uniquely upregulated in Xu22 under salt stress. In addition, 104 upregulated and 112 downregulated DEPs were identified in Xu32 compared with Xu22 under salt stress. Among them, the expression of many proteins, such as elongation factor, catalase, DEAD-box ATP-dependent RNA helicase, rubisco activase, glutamate dehydrogenase, and ATP-citrate synthase was upregulated in Xu22 vs Xu32 under salt stress (**Supplementary Table S7**).

**Figure 3:**
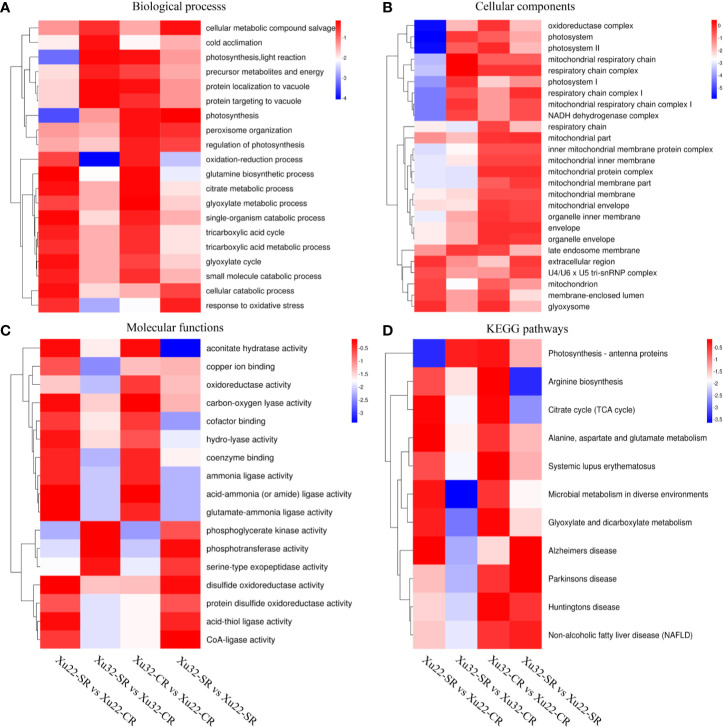
Heatmaps of the enriched GO classifications and KEGG pathways of differentially expressed proteins (DEPs) in Xu22 and Xu32 in proteome analysis. **(A–C)** The biological process (BP, **A**), cellular component (CC, **B**), and molecular function (MF, **C**) of the GO classifications of the annotated DEPs. **(D)** Enriched KEGG pathways of the DEPs in Xu22 and Xu32. The color scales at right indicate the relative expression. Red and blue shading indicate the relative high or low expression levels, respectively.

GO annotations of the DEPs displayed that there were multiple categories such as “glutamine biosynthetic process,” “single-organism catabolic process,” and “cellular catabolic process” in Xu22; “photosynthesis, light reaction” and “protein localization to vacuole” in Xu32 were remarkably enriched under the biological process after exposure to salt stress. And “photosynthesis,” “response to oxidative stress,” and “cellular metabolic compound salvage” were significantly enriched in Xu32 vs Xu22 under salt stress (**Figure 3A**). As for the celelular components, multiple terms were downregulated in Xu32 compared with Xu22 under salt stress, such as “respiratory chain,” “late endosome membrane,” and “membrane-enclosed lumen” (**Figure 3B**). It is interesting to note that many terms in the molecular function category were prominently enriched in Xu22, while only several terms were enriched in Xu32 under salt stress (**Figure 3C**). In addition, the enrichment analysis of DEPs based on KEGG database showed that many categories, including “citrate cycle”; “alanine, aspartate, and glutamate metabolism”; “microbial metabolism in diverse environments”; and “Alzheimer’s disease” were remarkably enriched in Xu22 under salt stress. However, only “photosynthesis-antenna proteins” was enriched in Xu32 under salt stress. In addition, “arginine biosynthesis” and “citrate cycle” were significantly downregulated in Xu32 compared with Xu22 under salt stress (**Figure 3D**).

To obtain more information on response changes under salt stress, the identified proteins were matched with the genes from RNA-seq analysis. Of the 3,123 identified proteins, 1,113 had corresponding genes in the RNA-seq data (**Supplementary Table S8**). The results showed that the correlation between gene and protein expression was very weak, only a few genes and their corresponding proteins have consistent expression profiles. For instance, 8 and 11 DEGs showed consistent expression between the transcription level and translation level in Xu22 and Xu32 under salt stress, respectively. And the encoding products of common upregulated genes between transcriptome and proteome analysis included aldehyde dehydrogenase, glutamate dehydrogenase, and glutamine synthetase in Xu22 compared with Xu32 under salt stress (**Supplementary Table S9**). However, 68 and 144 proteins was differentially expressed in Xu22 and Xu32 under salt stress, respectively, but no changes in the expression of their corresponding genes were detected. 126 and 77 proteins were differentially expressed in Xu32 vs Xu22 under salt stress and Xu32 vs Xu22 without salt stress, respectively, while no changes in their gene expression were observed. Similarly, 20 and 30 DEGs was detected in Xu22 and Xu32 under salt stress, respectively, while no corresponding DEPs were identified (**Supplementary Table S9**).

### Identification of Salt-Responsive TFs in Sweet Potato and Overexpression of *IbNAC7* Improved Salt Tolerance in Transgenic *Arabidopsis*

The identification and characterization of stress-responsive TFs is crucial for the development of transgenic crops with improved stress tolerance. In this study, a total of 1,618 differentially expressed TFs were identified with FDR < 0.01 and |log2 (fold change)| > 1 (**Supplementary Table S10**). Representative differentially expressed TFs were shown in **Table 1**, including various salt tolerance/stress-related bZIP, bHLH, ERF, MYB, NAC, and WRKY TFs, and their diverse transcription profiles suggest their pivotal regulatory roles in salt stress response. Wherein, MYB (98 members), WRKY (95 members) and NAC (74 members) are the three TF families with the largest number of differential expression. Interestingly, the amount of upregulated expressed TFs detected in salt-tolerant Xu22 was significantly lower than that in salt-sensitive Xu32 under salt stress (**Table 1**), the specifically upregulated TFs in Xu22 may make a positive contribution to its salt tolerance. Previously, 12 stress-responsive *IbNAC* genes were selected based on the present RNA-seq data. Among them, the transcription of *IbNAC7* (one of the 74 NACs) was remarkably upregulated by multiple abiotic stresses and hormones, such as salt, cold, ABA, and ACC (Meng et al., 2018), indicating that *IbNAC7* may be involved in the stress response of sweet potato.

**Table 1:** Representative salt stress-responsive TFs under salt stress in sweet potato transcriptome analysis.

Name	Total No.	Xu22-SR vs Xu22-CR		Xu32-SR vs Xu32-CR		Xu22-CR vs Xu32-CR		Xu22-SR vs Xu32-SR	
		Upregulated	Downregulated	Upregulated	Downregulated	Upregulated	Downregulated	Upregulated	Downregulated
ARF	22	6	4	6	3	3	10	1	5
bHLH	34	5	10	6	9	5	9	2	12
bZIP	15	2	0	5	3	3	3	2	3
ERF	32	12	4	13	2	6	8	4	14
HSF	25	7	3	16	2	4	3	0	5
MADS	16	3	1	2	3	4	6	2	7
MYB	98	34	19	35	22	14	19	5	22
NAC	74	36	1	39	4	10	13	11	21
GRAS	17	4	1	5	3	2	5	2	6
SBP	4	0	0	1	0	0	2	1	2
TCP	9	3	3	0	2	0	3	1	1
Trihelix	6	1	0	1	0	1	2	1	3
Whirly	3	0	0	0	0	2	1	1	1
WRKY	95	43	9	44	10	9	16	20	23
zinc finger	49	3	7	7	14	10	12	5	18

CR, control fibrous roots; SR, salt-treated fibrous roots.

**Figure 4:**
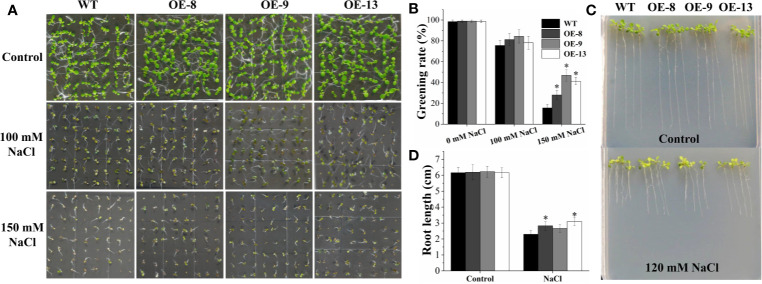
The germination greening rate and root length of transgenic plants overexpressing *IbNAC7* were improved under salt stress. **(A**, **B)** Comparisons of germination phenotype **(A)** and germination greening rate **(B)** between WT and transgenic seeds (n ≥ 60 each) grown on 1/2 MS medium containing 0, 100, and 150 mM NaCl for 10 d. **(C, D)** Comparisons of growth phenotype **(C)** and root length **(D)** between WT and transgenic seedlings (n ≥ 20 each) grown on 1/2 MS medium with or without NaCl for 10 d. Data are the means ± SE of three independent biological experiments. Asterisks indicate statistical signiﬁcance (*P < 0.05) between the WT and transgenic plants.

Subsequently, eight transgenic *Arabidopsis* lines overexpressing the *IbNAC7* gene were obtained, and the results displayed that all the transgenic lines showed remarkably higher transcription of *IbNAC7* than that in WT plants (**Supplementary Figure S8**). Three T3 homozygous lines with high expression of *IbNAC7* were selected for salt tolerance test. Firstly, the salt tolerance of transgenic lines was examined at the germination and post-germination stages. No obvious differences in germination greening rates on 1/2 MS medium with 0 and 100 mM NaCl between transgenic and WT seeds were observed. However, the germination greening rates of transgenic lines were notably higher than that of WT plants under 150 mM NaCl conditions (**Figures 4A, B**). And the transgenic and WT seedlings showed similar growth on control medium, while the transgenic lines provided remarkably longer roots than that of WT under 120 mM NaCl stress (**Figures 4C, D**). The results showed that *IbNAC7* conferred salt tolerance during the germination and post-germination stages of *Arabidopsis*.

### Performance of Transgenic *IbNAC7* Plants Under Salt Stress in Soil and Salt Tolerance Evaluation

**Figure 5:**
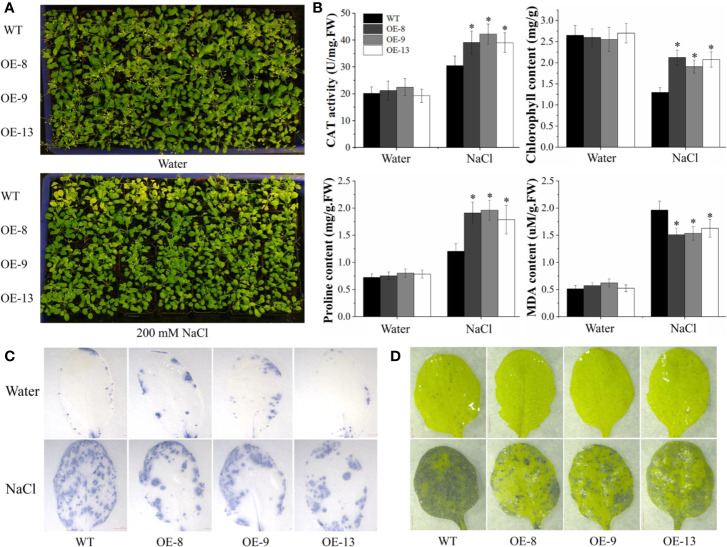
Comparisons of WT and transgenic plants overexpressing *IbNAC7* treated with water or salt stress in soil. **(A)** Phenotype comparison of WT and transgenic plants exposed to salinity stress in soil. 20-d-old plants were randomly selected for the salt tolerance assay by irrigating with 200 mM NaCl solution every 3 d from the bottom of pots. 15 plants from each line were employed for one experiment, and three independent experiments were conducted. **(B)** Comparisons of CAT activity, chlorophyll, proline and MDA content between WT and transgenic plants under normal and salt stress conditions. Data are the means ± SE of three independent biological experiments. Asterisks indicate statistical signiﬁcance (*P < 0.05) between the WT and transgenic plants. **(C, D)** Histochemical staining of TB **(C)** and NBT **(D)** of transgenic *Arabidopsis* plants under under normal and salt treatment for 15 d.

The performance of transgenic lines under NaCl stress was further tested in soil, 20-d-old WT and transgenic lines were irrigated with 200 mM NaCl every 3 d. Under control conditions, normal morphological phenotypes were observed in WT and transgenic lines. Nevertheless, transgenic lines displayed better growth after salt stress, such as delayed leaf necrosis and yellowing at 15 d post-treatment (**Figure 5A**). To characterize the salt tolerance of the transgenic lines, several stress-related physiological parameters were detected. No significant differences in the physiological analysis between WT and transgenic lines were observed under normal conditions. After 15 d of salt stress, the CAT and SOD activity and chlorophyll content of transgenic plants were markedly higher than those of WT. In contrast, transgenic plants accumulated less MDA than the WT plants (**Figure 5B**). Besides, biochemical staining was analyzed by NBT and TB using the detached leaves. In the absence of salt stress, transgenic and WT plants displayed similar basal levels of ROS and cell death. After exposure to salt stress for 15 d, transgenic lines accumulated much less ROS and dead cells than that of WT plants (**Figures 5C, D**). These results suggested that transgenic *Arabidopsis* overexpressing *IbNAC7* had significantly improved salt tolerance compared to the WT plants.

### IbNAC7 Affect Multiple Groups of Biotic- and Abiotic Stress-Related Genes Under Salt Stress

**Figure 6:**
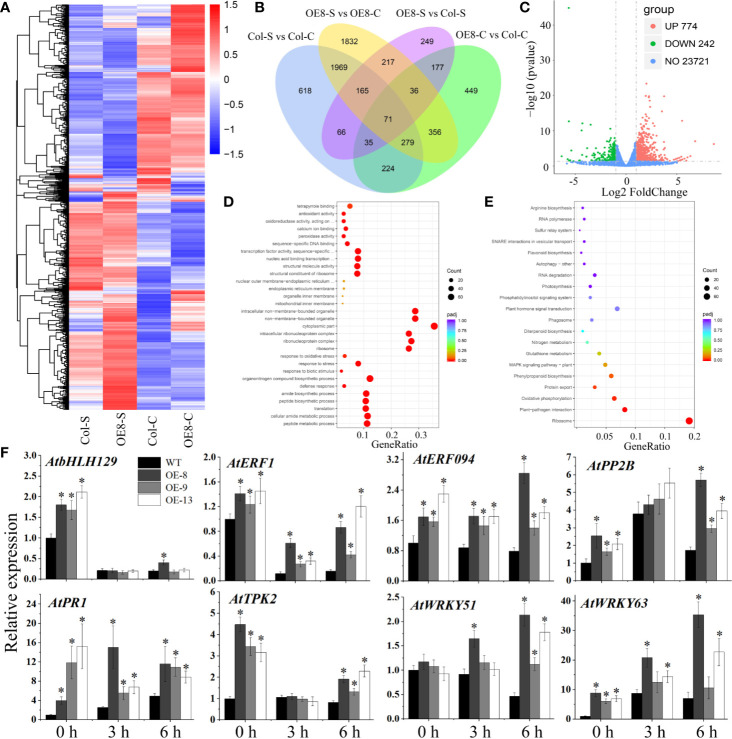
RNA-seq analysis of stress-related genes affected by IbNAC7 under salt stress. **(A)** The DEGs were hierarchically clustered according to the Log_10_ (FPKM+1) values. The red and blue bands represent induced and repressed DEGs, respectively. **(B)** Venn diagrams between WT and transgenic plants suggested speciﬁc DEGs and shared DEGs under control and salt stress conditions. Overlapping region indicates co-expressed DEGs between WT and transgenic plants. The numbers only in one circle represent DEGs expressed only in WT or transgenic plants. **(C)** Volcano plot of DEGs in OE-8 vs WT plants after salt treatment. The red and green dots represent the upregulated and downregulated DEGs, respectively, and the blue dots represent non-DEGs. **(D, E)** GO classifications **(D)** and KEGG pathways **(E)** of annotated upregulated genes in OE-8 vs WT plants under salt stress. X-axis and Y-axis represent the GeneRatio and the category names, respectively. Coloring correlates with the padj. The lower the padj, the more significant the enrichment. The point size correlates with the number of DEGs. **(F)** Validation of the expression of selected stress-related genes by qRT-PCR between WT and transgenic plants under normal and salt stress conditions. 10-d-old plants were soaked in liquid 1/2 MS medium containing 100 mM NaCl solution for 3 and 6 h, and then the whole plants before and after NaCl treatment were collected. The relative expression levels were normalized to 1 in WT plants at 0 h. Data are the means ± SE of three independent biological experiments. Asterisks indicate statistical signiﬁcance (*P < 0.05) between the WT and transgenic plants.

To clarify the potential mechanism of salt tolerance modulated by IbNAC7, RNA-seq was used to detect the gene expression differences of transgenic plants (line 8) under NaCl stress. A summary of the sequencing assembly is shown in **Supplementary Table S11**. At least 94.31% of the genes in each library were mapped to the *Arabidopsis* genome, and the RNA data displayed a strong expression correlation (R^2^ ≥ 0.791). Venn diagram showed that 2,441 and 943 DEGs were specifically expressed in transgenic and WT plants under salt stress, respectively, and 2,484 DEGs were expressed in both plants (**Figures 6A, B**). A total of 1016 DEGs, including 774 upregulated and 242 downregulated genes, were detected in the transgenic lines compared with those in WT plants under salt stress (**Figure 6C**, **Supplementary Table S12**). Representative upregulated DEGs included 306 salt tolerance/stress-related TFs, such as AP2/ERF, bHLH, MYB, NAC, WRKY, and Zinc finger protein, indicating their vital roles under salt stress. In addition, the upregulated genes are also associated with diverse stress response. For instance, multiple upregulated genes encoded ABC transporter family proteins, LEA proteins, pathogenesis-related proteins, peroxidases, and PPR proteins (**Supplementary Table S12**).

When the DEGs were analysed for GO annotations, multiple stress-related GO terms were found, such as the peroxidase activity, antioxidant activity, defense response, and response to biotic stimulus (**Supplementary Figure S9A**). And multiple terms including response to stress, response to oxidative stress, response to biotic stimulus were significantly upregulated in transgenic lines compared with WT plants under salt stress (**Figure 6D**). Moreover, DEG-associated KEGG pathways were also identified. The results showed that the pathways related to ribosome, carbon metabolism, and starch and sucrose metabolism were significantly enriched in transgenic plants compared with those in WT plants under salt stress (**Supplementary Figure S9B**). And the pathways related to ribosome, plant-pathogen interaction, and phenylpropanoid biosynthesis were remarkably upregulated in transgenic plants (**Figure 6E**). Taken together, these results suggested that IbNAC7 is involved in the regulation of numerous and diverse stress-related genes in response to salt stress in *Arabidopsis*.

To validate the DEGs obtained from RNA-seq data, the expression levels of eight genes were detected by qRT-PCR. The upregulated DEGs were selected being representative of important genes in stress response. For instance, TF encoding genes *AtbHLH129* (Tian et al., 2015), *AtERF1* (Lorenzo et al., 2003), *AtERF094* (Zarei et al., 2011), *AtWRKY51* (Yan et al., 2018) and *AtWRKY63* (Ren et al., 2010), K^+^ channel protein encoding gene *AtTPK2* (Isayenkov and Maathuis, 2013), stress-related F-box protein encoding gene *AtPP2B2* (Stefanowicz, 2015) and pathogenesis-related gene *AtPR1* (Pecenkova et al., 2017). Our results showed that the expression of almost all the selected genes was significantly upregulated in transgenic lines compared to that of WT under both control and salt conditions, which was consistent with that obtained by RNA-seq analysis, although there were variations in fold changes among the transgenic lines (**Figure 6F**). Therefore, these results demonstrated that IbNAC7 may affect the transcription of multiple groups of stress-related genes under salt stress.

## Discussion

pt?>Cultivating salt tolerant and high-yielding crop varieties is the most effective way to reduce crop yield losses. Therefore, it is critical to understand the salt stress response and tolerance mechanism of plants, which will help us to improve the stress tolerance of plants through molecular breeding and transgenic approaches. Genome-wide detection of specific stress-responsive genes based on transcriptome analysis in many crops with different characteristics has been increasingly conducted under various stresses. For instance, the gene expression dynamics of two contrasting genotypes, such as rice, sesame, cotton, and maize, under salt-treated and normal conditions were examined, and large numbers of diverse stress-related genes were identified (Guo et al., 2015; Li et al., 2018; Wang M. et al., 2019; Zhang et al., 2019). However, there is a lack of research on sweet potato, a hexaploid heterozygous non-model crop, responses to salt stress, and the regulations at translational levels have been rarely studied. In addition to RNA-seq analysis, proteome analysis is also critically necessary for understanding the change in translational regulation during stress responses. In this study, comparative transcriptome and proteome analysis of two contrasting sweet potato cultivars with different salt tolerance were investigated, which will provide a unique opportunity to gain insights into the candidate genes and proteins involved in salt stress response in this important crop. To our knowledge, this is the first comparison of salt stress-responsive transcriptome and proteome in sweet potato with contrasting genotypes.

In the present study, distinct differences were detected in transcription and translation levels between the two sweet potato genotypes even without salt stress. A total of 16,396 DEGs and 724 DEPs were identified under normal and salt-treated conditions, suggesting that transcription and translation regulations play a crucial role in the response of sweet potato to salt stress. The results showed that the correlation between gene and protein expression of both genotypes was very weak. Similar to our previous report (Dong et al., 2019) and multiple other studies (Bogeat-Triboulot et al., 2007; Liu et al., 2016; Li et al., 2018), the results showed that not all mRNA: protein ratios reflected the corresponding changes in transcription and protein levels (Haider and Pal, 2013). This may be due to the technical limitations of the proteome method or the possible occurrence of posttranscriptional regulation during salt stress response in sweet potato, making it difficult to compare with RNA-seq data. In view of this, we mainly focus on the discussion of transcriptome data between the two genotypes. Transcriptome data showed that many significantly enriched functional GO and KEGG terms of DEGs are consistent between Xu22 and Xu32 under salt stress, indicating that the main salt stress response pathways between the two cultivars may be similar. However, the “ribosome” and “glutathione metabolism” pathways in Xu22 were significantly upregulated compared to Xu32, and “N-Glycan biosynthesis” and “glucosinolate biosynthesis” were only enriched under salt stress in Xu22. These pathways be involved in the regulation of the salt tolerance of Xu22. Differently, the methionine metabolism pathway was previously shown as a primary contributor to the salt-tolerant jute (Yang et al., 2017). In addition, in proteome analysis, we also found that many proteins were common upregulated in the two genotypes under salt stress, such as peroxidase, elongation factor, phosphoglycerate kinase, plasma membrane ATPase, and NADH dehydrogenase. However, multiple proteins such as cytochrome P450, cinnamic acid 4-hydroxylase, and lipoxygenase were uniquely upregulated in Xu22 under salt stress, suggesting that these specific proteins may affect the contrasting salt tolerance between the two genotypes. Besides, previous reports showed that the numbers of upregulated genes of salt-tolerant varieties were higher than that of salt-sensitive varieties under salt stress (Geng et al., 2019; Zhang et al., 2019). However, our current RNA-seq data showed that the numbers of upregulated and downregulated genes in salt-sensitive Xu32 were more than that in salt-tolerant Xu22. And 1,764 and 3,454 DEGs were specifically detected in Xu22 and Xu32 under salt stress, respectively. Especially, many genes encoding stress-related proteins/factors, including NAC TF, WRKY TF, ERF TF, PPR protein, F-box protein, zinc finger protein, potassium transporter, methyltransferase, cytochrome P450 were uniquely expressed or significantly upregulated in salt-tolerant Xu22 under salt stress. Therefore, these differential genes and pathways may contribute to the difference in salt tolerance between the two contrasting genotypes, and the salt tolerance of Xu22 could be enhanced by high expression of some genotype-specific genes.

Salt stress has primary osmotic shock and ion-toxicity effects, while secondary effects are complex, including reactive oxygen species (ROS) burst, cell component damages, and metabolic dysfunctions in plant cells (Munns and Tester, 2008; Zhu, 2016). During the initial phase, cell expansion, cell wall and protein biosynthesis, and photosynthetic activity of plant cells are all inhibited, and many plants can accumulate compatible solutes and ABA to preserve the osmotic pressure (Apel and Hirt, 2004). At the same time, the ratios of Na^+^/K^+^ and Na^+^/Ca^2+^ are also altered (Apse and Blumwald, 2007). In this context, increased expression of many genes related to cell division, amino acid metabolism, sucrose synthesis, photosynthetic activity, ABA signaling as well as potassium and potassium transport were observed in Xu22 and Xu32 under salt stress. This is consistent with the previous observations on salt-induced accumulations of special metabolites in multiple plants such as rice, sesame, and sugar beet (Wang et al., 2018; Geng et al., 2019; Zhang et al., 2019). Accordingly, KEGG enrichment analysis of the DEGs showed that “phenylpropanoid biosynthesis,” “starch and sucrose metabolism,” “biosynthesis of amino acids,” “flavonoid biosynthesis,” and “plant hormone signal transduction” categories were significantly enriched. Similar metabolism enrichment was also enhanced in salt-tolerant sesame (Zhang et al., 2019). Enhanced synthesis of polyphenols, such as phenolic acids and flavonoids are detected under multiple abiotic stresses, and they have the potential to scavenge ROS, which can help plants respond to environmental stimuli (Sharma et al., 2019). Plant hormones including ABA, SA, JA, and ethylene play critical roles in regulating plant response to extensively biotic and abiotic stresses (Bari and Jones, 2009). Besides, amino acids can function as compatible solutes and the precursors of secondary metabolites can protect plants from various stresses, suggesting that amino acid metabolisms play a pivotal role in plant response to stress (Stepansky and Galili, 2003; Haeusler et al., 2014). The results suggested that many functional classifications of DEGs were similar between salt-tolerant Xu22 and salt-sensitive Xu32. Particularly, several categories were preferably enriched in Xu22, such as “N-Glycan biosynthesis” and “glucosinolate biosynthesis,” indicating that these categories might play a role in the differential salt tolerance between Xu22 and Xu32. The later phase is mainly associated with ROS, and the imbalance between ROS production and ROS scavenging will lead to subsequent oxidative stress (Xu et al., 2018). To maintain ROS homeostasis under adverse stress, plants have evolved multiple antioxidant mechanisms, including a ROS scavenging system. In our present study, the data revealed that numerous DEGs in both Xu22 and Xu32 were associated with antioxidant activity and peroxisome under salt stress, implying that ROS scavenging-related antioxidant metabolisms are vital tolerance mechanisms for sweet potato adaptive response to salinity stress. Therefore, our data strongly suggested that salt-induced accumulations of these pivotal metabolites through biosynthesis or metabolism pathways may contribute to enhancing salt tolerance of sweet potato.

TFs are critical components that regulate plant signal transduction and gene expression in response to various biotic and abiotic stresses (Erpen et al., 2018; Zhu et al., 2018; Li et al., 2019). In our transcriptome data, a total of 1,618 differentially expressed TFs were examined, including bZIP, bHLH, ERF, MYB, NAC, and WRKY, suggesting their important roles in regulating the salt tolerance of sweet potato. Interestingly, the amount of upregulated TFs detected in salt-tolerant Xu22 was lower than that in salt-sensitive Xu32 under salt stress. Besides, the differential expression of TFs of the same family suggests that different members may have distinct biological functions or regulatory mechanisms in sweet potato response to salt stress. NACs are promising candidate factors for genetic engineering to improve crop tolerance, which have been extensively demonstrated by numerous stress-responsive NACs in various plant species (Mohanta et al., 2020). A total of 74 NACs were differentially expressed in the two phenotypes under salt stress, and the transcription of *IbNAC7* (one of the 74 NACs) was remarkably induced by salt, cold, ABA, and ACC treatments (Meng et al., 2018), suggesting that *IbNAC7* may be involved in the response to environmental cues. Subsequently, salt tolerance test of transgenic *Arabidopsis* overexpressing *IbNAC7* suggested this gene played important roles in salt tolerance. Similarly, numerous transgenic plants achieved by overexpression of *OsNAC6* (Nakashima et al., 2007), *ONAC022* (Hong et al., 2016), and *ThNAC13* (Wang et al., 2017b) displayed significantly improved salt tolerance. Our previous reports also showed that *SlNAC4* and *SlNAC11* participated in the regulation of tomato salt and drought tolerance (Zhu et al., 2014; Wang et al., 2017a). The transgenic *Arabidopsis* overexpressing *IbNAC7* not only displayed morphological advantages in germination greening rate and root length, but also displayed higher CAT activity, chlorophyll, and proline contents than that of WT under salt stress. CAT is the key antioxidant enzyme involved in ROS scavenging (Gill and Tuteja, 2010). Chlorophyll, the main pigment of plant photosynthesis, was reported that its content is positively correlated with salt tolerance (Zhu et al., 2018). Proline functions as a regulator of antioxidant system to stabilize proteins (Hong et al., 2016). In addition, the reduced MDA content and histochemical staining suggested that ROS accumulation in transgenic plants was less than than that in WT. Similarly, previous reports showed that overexpression of *NAC57* and *OsNAC2* could preclude excess ROS accumulations and improve salt tolerance in *Arabidopsis* (Mao et al., 2018; Yao et al., 2018). These results suggest that transgenic plants may have more robust photosynthetic capacity and less oxidative damage than WT plants, thus helping them to enhance tolerance to salt stress.

Besides, the improved salt tolerance of transgenic plants was also characterized by the upregulated expression of numerous and diverse types of downstream stress regulators compared to that in WT plants. A total of 1016 DEGs were detected by RNA-seq in transgenic plants compared with those in WT plants under salt stress. GO annotations of the DEGs showed that multiple terms, such as response to stress, response to oxidative stress, response to biotic stimulus were significantly upregulated in transgenic lines compared with WT plants under salt stress. qRT-PCR validation analysis showed that the expression of multiple stress-related genes in transgenic lines was indeed upregulated. For instance, the TF encoding genes *AtbHLH129*, *AtERF1*, *AtERF094*, *AtWRKY51*, and *AtWRKY63*. Wherein, *AtWRKY63* plays an important role in the response of *Arabidopsis* to ABA and drought stress (Ren et al., 2010) and *AtERF1* integrates signals from ethylene and jasmonate pathways in plant defense (Lorenzo et al., 2003). In addition, pathogenesis-related AtPR1 is an important defense protein in *Arabidopsis* (Pecenkova et al., 2017) and K^+^ channel protein AtTPK2 was reported to complement the K^+^ uptake deficient *E. coli* mutant (Isayenkov and Maathuis, 2013), both of their expression was significantly upregulated in transgenic plants. The enhanced expression of these genes may lead to alterations in biochemical and physiological pathways, which are important for *Arabidopsis* to adapt to salt stress. Taken together, these results suggested that IbNAC7 may be involved in the regulation of numerous and diverse stress-related genes in *Arabidopsis* responses to salt stress.

Collectively, in this study, comparative transcriptome and proteome analyses were simultaneously conducted to investigate the salt tolerance mechanisms between salt-tolerant Xu22 and salt-sensitive Xu32. We have shown that the tolerant and sensitive sweet potato genotypes response differently to salt stress, and large amounts of DEGs and DEPs have been detected in the two cultivars, suggesting that transcription and translation regulations play a crucial role in the response of sweet potato salt stress. Importantly, our results showed that overexpression of *IbNAC7* remarkably enhanced salt tolerance in *Arabidopsis* mainly by deterring the accumulation of ROS. In addition, the data also provide numerous valuable candidate genes that may facilitate the functional characterization of the salt-responsive genes, and many of which can be used to breed salt-tolerant sweet potato cultivars. Overall, these results provide a revealing insight into sweet potato molecular response to salt stress and underlie the complex salt tolerance mechanisms between genotypes, and *IbNAC7* has been shown as a promising candidate gene to enhance the salt tolerance of sweet potato.

## Data Availability Statement

The datasets generated for this study can be found in the NCBI database under the bioproject PRJNA631585 and PRJNA649852. The proteomics data can be found in FigShare https://doi.org/10.6084/m9.figshare.12758444.v1.

## Author Contributions

MZ and ZL designed the experiments. MZ and XM analyzed the data and wrote the manuscript. XM, SL, TD, and TX performed the experiments. DM and SP analyzed the data and improved the manuscript. All authors contributed to the article and approved the submitted version.

## Funding

This work was supported by National Natural Science Foundation of China (31700226), China Agriculture Research System (CARS-10-B3), and the Priority Academic Program Development of Jiangsu Higher Education Institutions (PAPD).

## Conflict of Interest

The authors declare that the research was conducted in the absence of any commercial or financial relationships that could be construed as a potential conflict of interest.

The reviewer SK declared a past co-authorship with several of the authors ZL and DM to the handling editor.
