# Supplementary material for: Comparative Transcriptome and Proteome Analysis of Salt-Tolerant and Salt-Sensitive Sweet Potato and Overexpression of IbNAC7 Confers Salt Tolerance in Arabidopsis
Source: Front Plant Sci. 2020 Aug 27;11:572540. doi: 10.3389/fpls.2020.572540 (PMC7481572; doi:10.3389/fpls.2020.572540)
Supplement: Supplementary file 1 [file DataSheet_1.docx]

**Title:**

**Comparative transcriptome and proteome analysis of salt-tolerant and salt-sensitive sweetpotato and overexpression of *IbNAC7* confers salt tolerance in *Arabidopsis***

**Authors:**

Xiaoqing Meng^1, 2^, Siyuan Liu^1, 2^, Tingting Dong^1, 2^, Tao Xu^1, 2^, Daifu Ma^1, 3^, Shenyuan Pan^1, 2^, Zongyun Li^1, 2*^, Mingku Zhu^1, 2*^

*1, Institute of Integrative Plant Biology, School of Life Science, Jiangsu Normal University, Xuzhou, China;*

*2, Jiangsu Key Laboratory of Phylogenomics & Comparative Genomics,School of Life Science, Jiangsu Normal University, Xuzhou, China;*

*3, Jiangsu Xuzhou Sweetpotato Research Center, Chinese Academy of Agricultural Sciences (CAAS), Xuzhou, China.*

**Authors for Correspondence:**

Mingku Zhu ([mingkuzhu007@126.com](mailto:mingkuzhu007@126.com)); Zongyun Li (zongyunli@jsnu.edu.cn)

Postal Address: School of Life Sciences, Jiangsu Normal University, 101 Shanghai Road, Xuzhou, Jiangsu Province 221116, China; Tel: 0086051683403172.


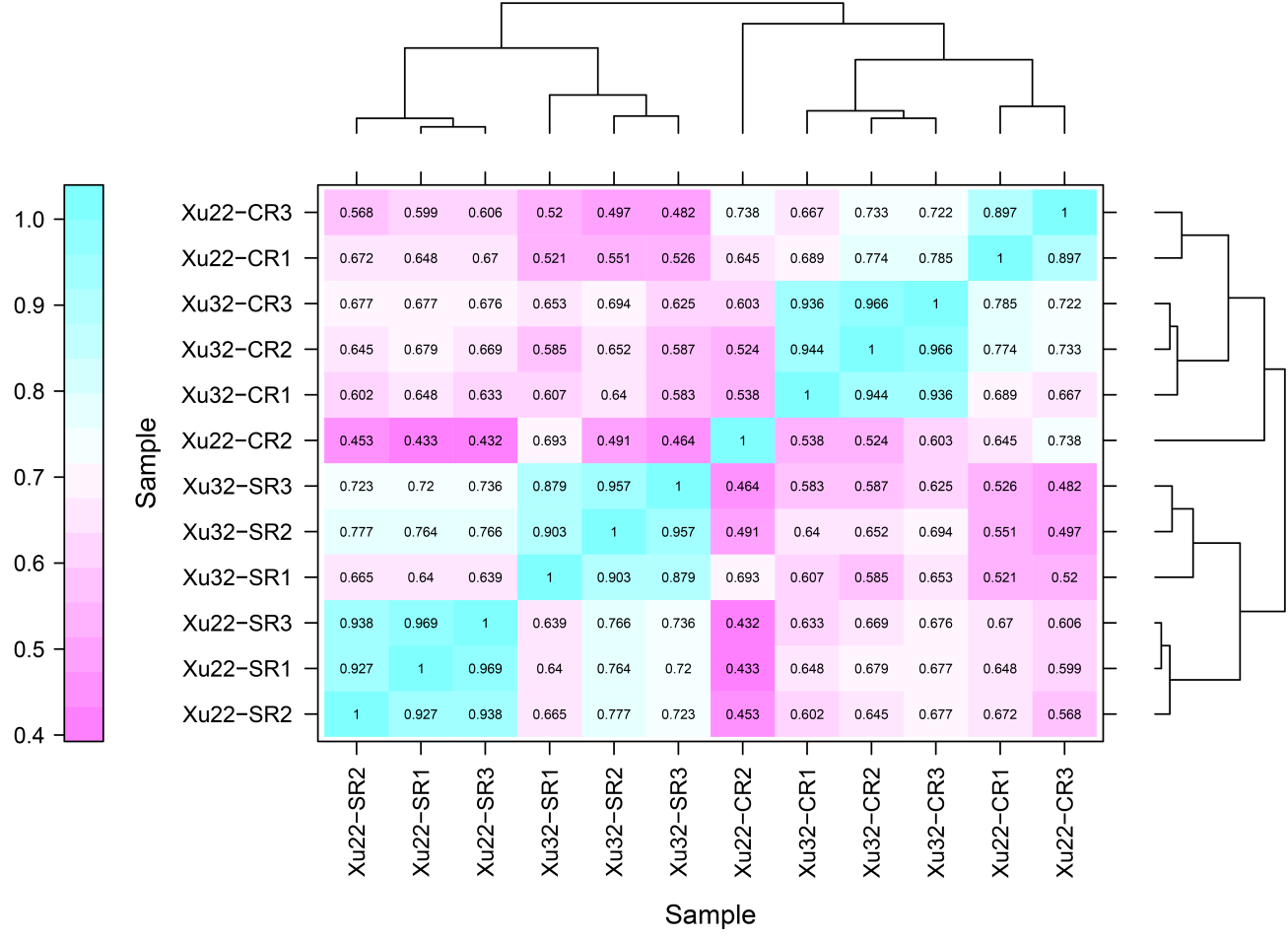
**Figure S**1. The pearson correlations among different samples in sweetpotato transcriptome analysis. CR: Control fibrous roots, SR: Salt-treated fibrous roots.


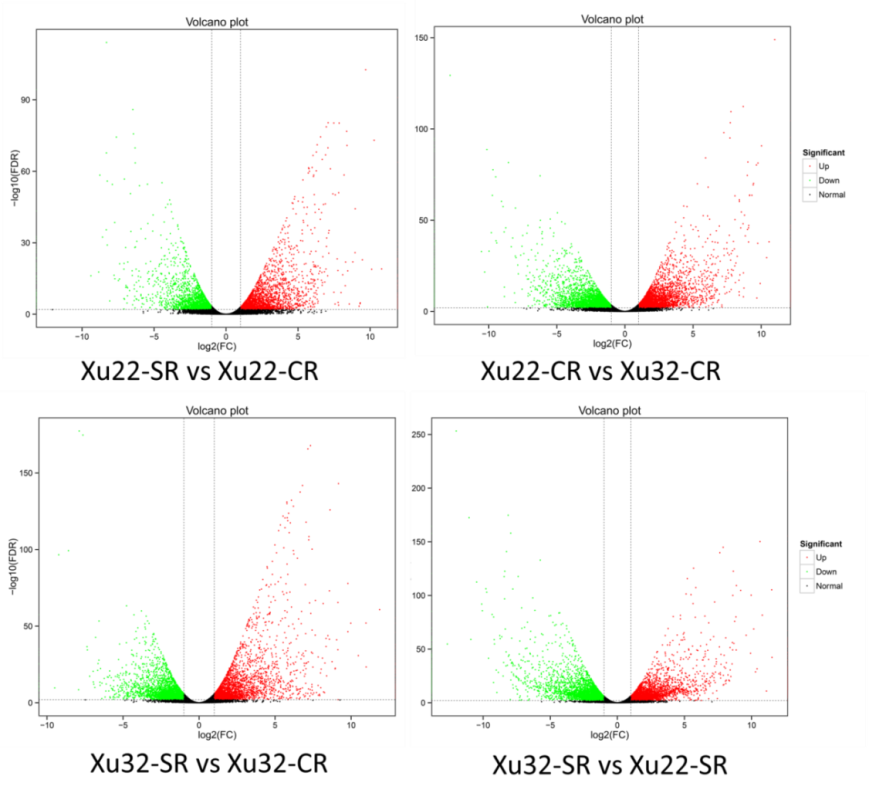
**Figure S**2. Volcano plots of DEGs among different samples under control and salt-treated conditions. Red and green dots represent the upregulated and downregulated DEGs, respectively, and black dots represent non-DEGs. CR: Control fibrous roots, SR: Salt-treated fibrous roots.


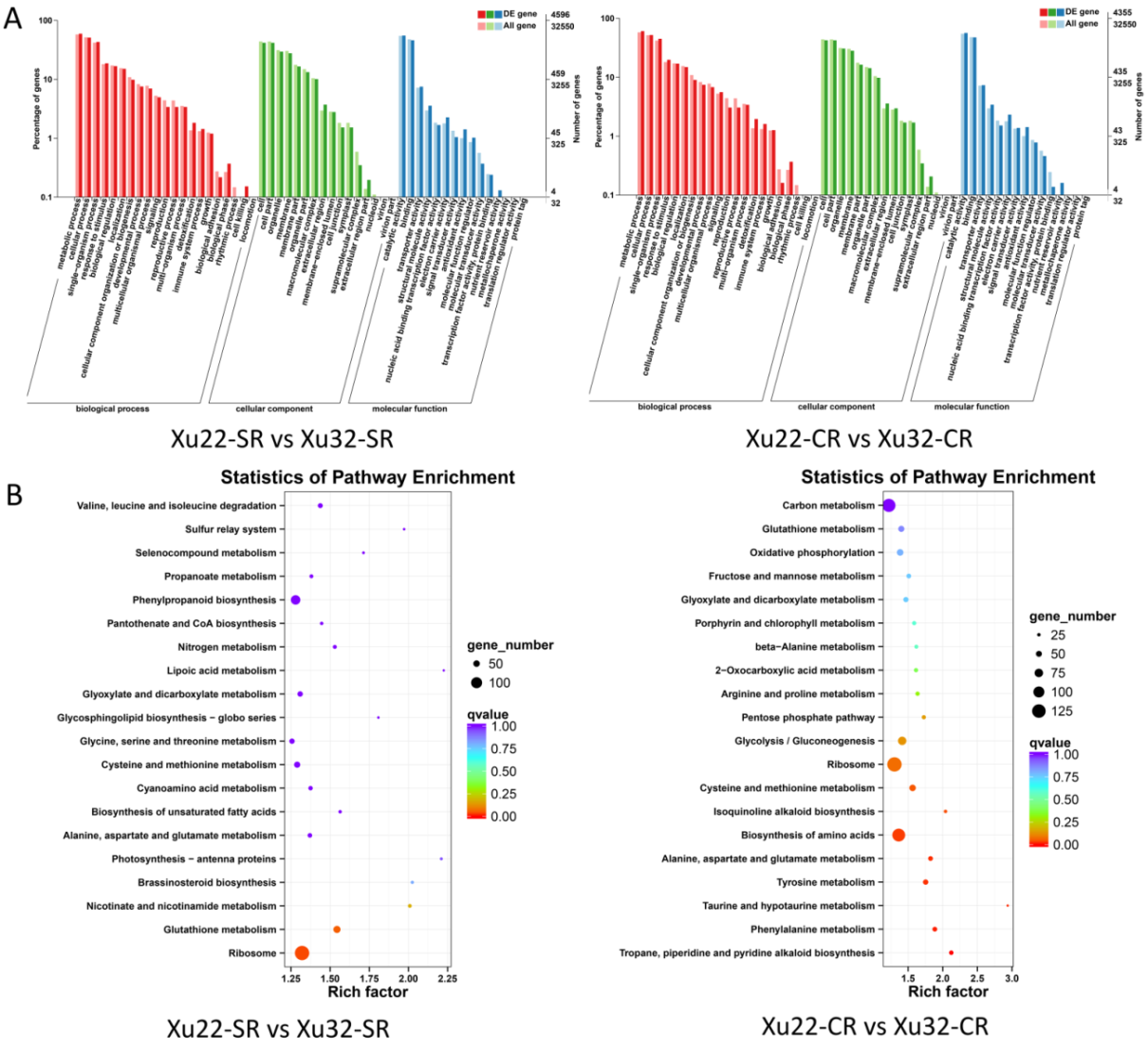
**Figure S3**. GO classifications and KEGG pathways of DEGs in Xu22 compared with Xu32 under control and salt-treated conditions. A. GO classification of annotated DEGs. The left Y-axis indicates the percentage of DEGs identified, and the right Y-axis indicates the number of DEGs. The DEGs were categorized based on the annotations of GO, and the numbers are displayed according to the biological process, cellular component, and molecular function. B. Enriched KEGG pathways of the DEGs. X-axis and Y-axis represent the GeneRatio and the terms of pathways, respectively. Coloring correlates with the q-value. The lower the q-value, the more significant the enrichment. Point size correlates with the numbers of DEGs. CR: Control fibrous roots, SR: Salt-treated fibrous roots.

**Figure S4**. KEGG pathways of the upregulated (A) and downregulated (B) genes among the different set of transcriptome data of sweetpotato. X-axis and Y-axis represent the GeneRatio and the terms of pathways, respectively. Coloring correlates with the q-value. The lower the q-value, the more significant the enrichment. Point size correlates with the numbers of DEGs. CR: Control fibrous roots, SR: Salt-treated fibrous roots.


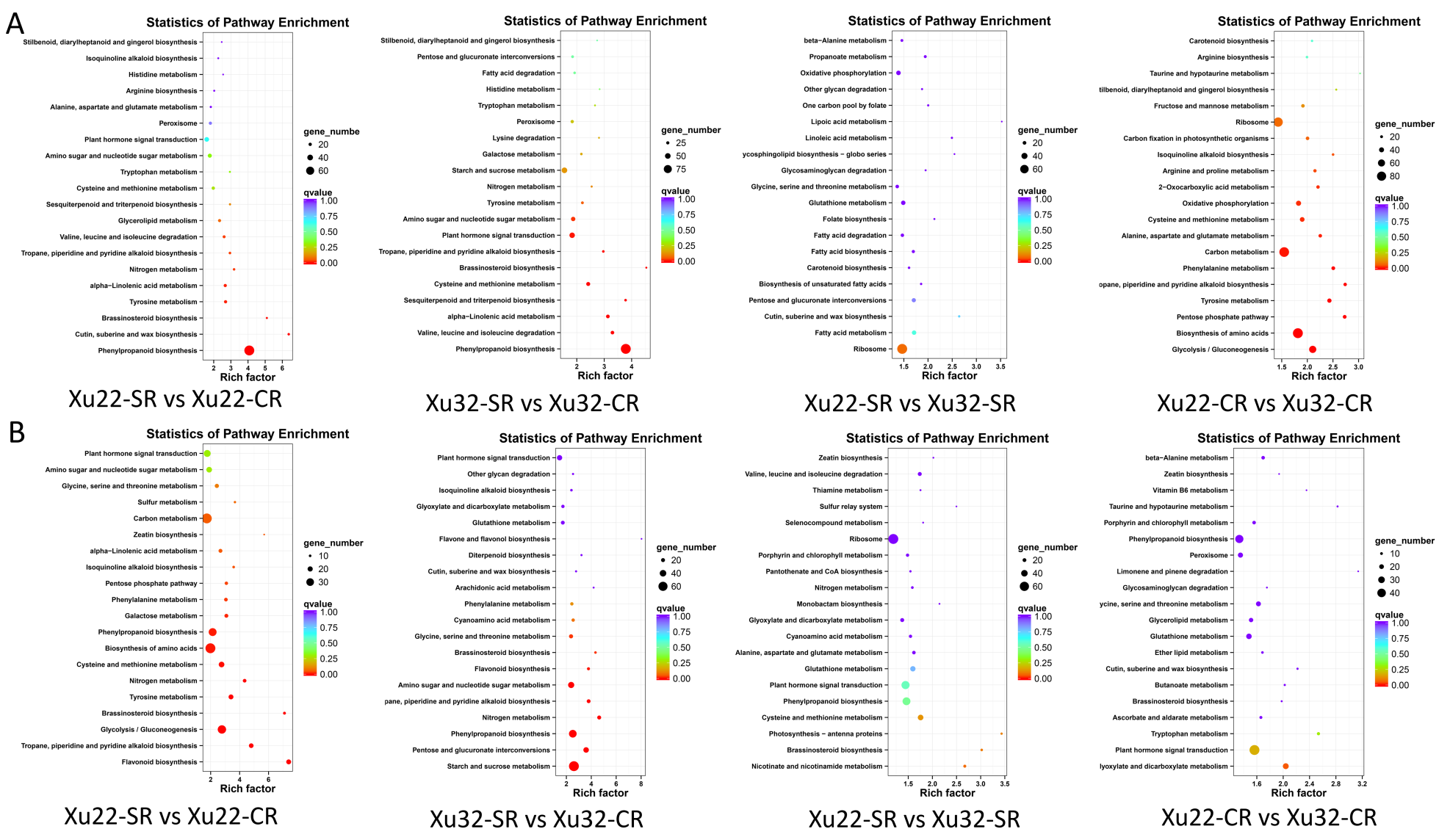


**Figure S**5. The pearson correlations among different samples in sweetpotato proteome analysis. CR: Control fibrous roots, SR: Salt-treated fibrous roots.


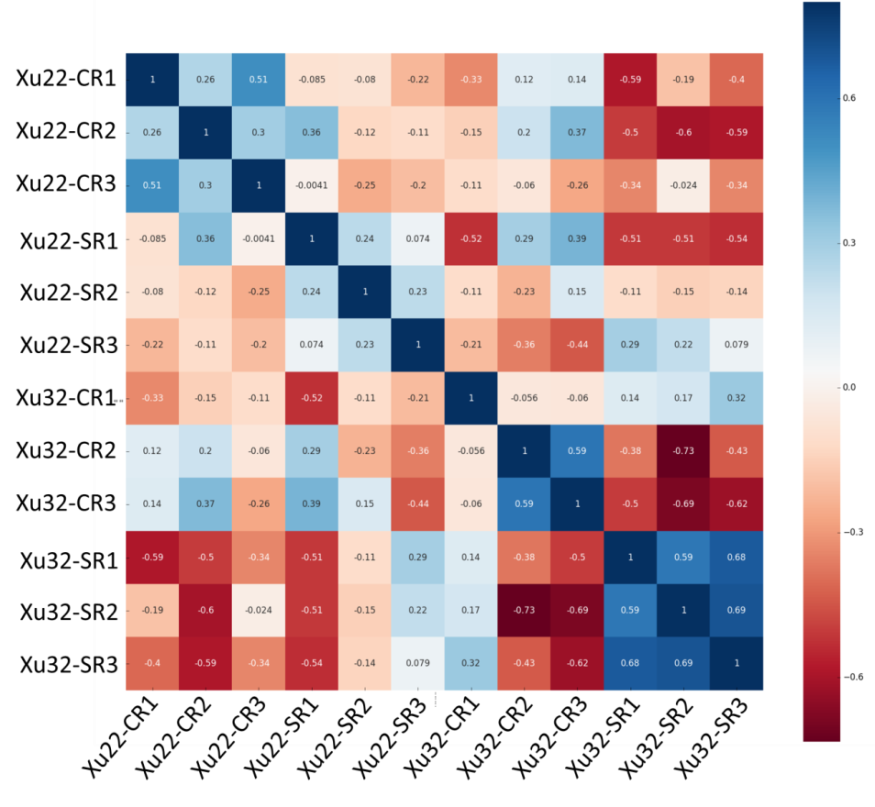


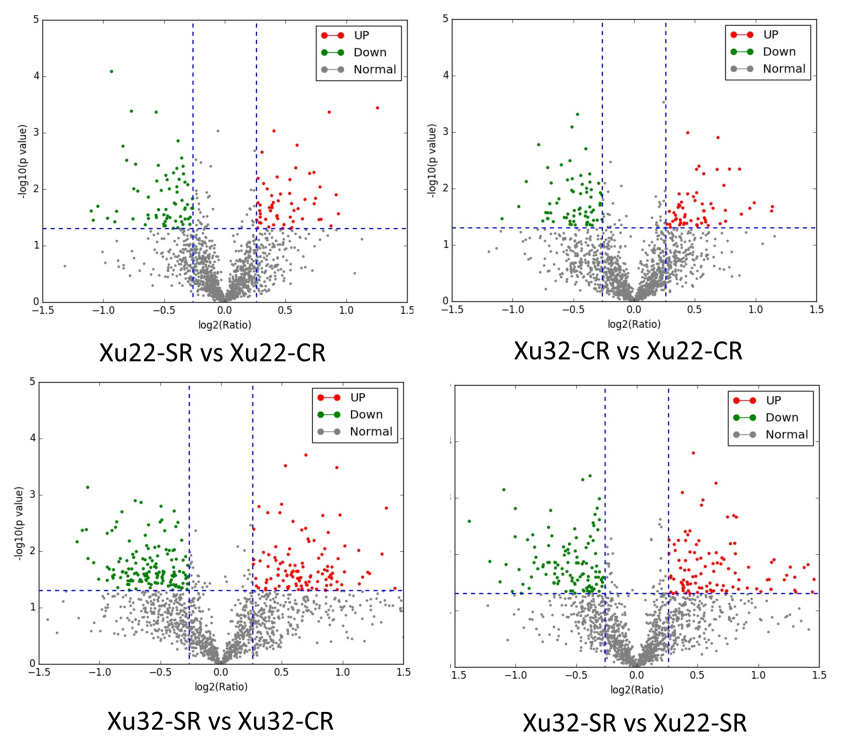
**Figure S**6. Volcano plots of DEPs among different samples under control and salt-treated conditions. Red and green dots represent the upregulated and downregulated DEPs, respectively, and black dots represent non-DEPs. CR: Control fibrous roots, SR: Salt-treated fibrous roots.


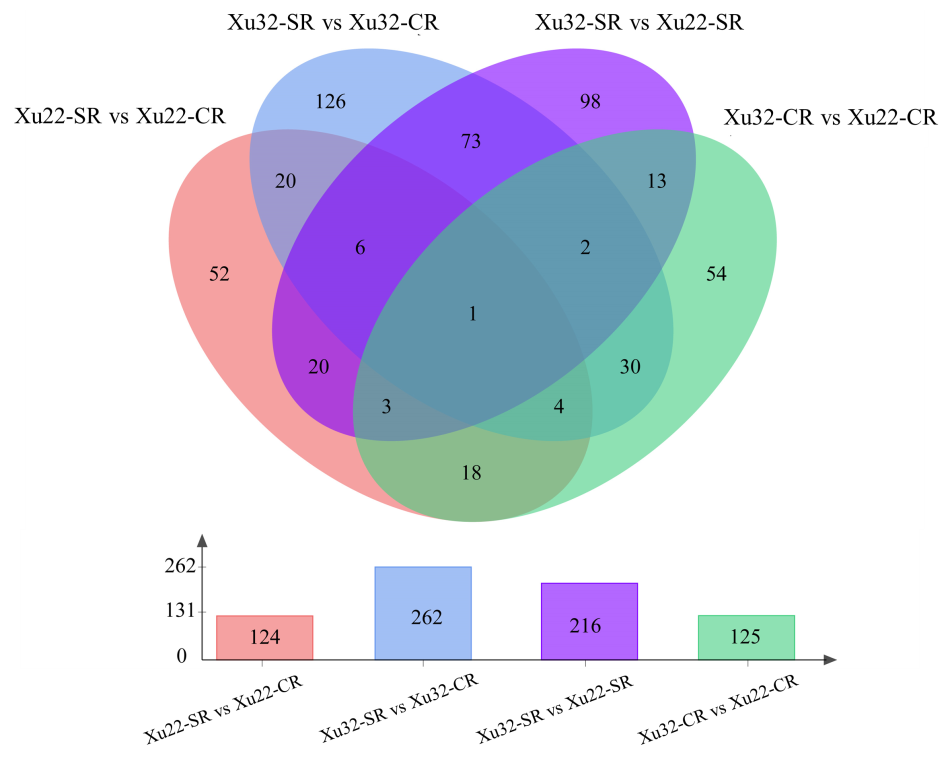
**Figure S**7. Venn diagrams in Xu22 and Xu32 suggesting that the DEPs were genotype-specific and salt-responsive. Overlapping regions indicate co-expressed DEPs among the different set of data, the numbers only in one circle represent DEPs that are only expressed in one library. CR: Control fibrous roots, SR: Salt-treated fibrous roots.

**Figure S8.** qRT-PCR analysis of the expression levels of *IbNAC7* in different transgenic lines. The relative expression was normalized to *AtEF1α*, and the expression was further calibrated using the transgenic line with the lowest *IbNAC7* expression. Data are the means ± SE of three independent biological experiments. Asterisks indicate statistical signiﬁcance (* P < 0.05) between the WT and transgenic plants.


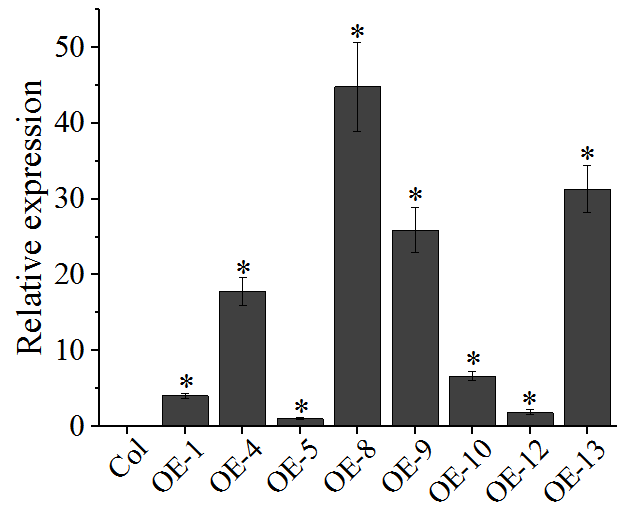


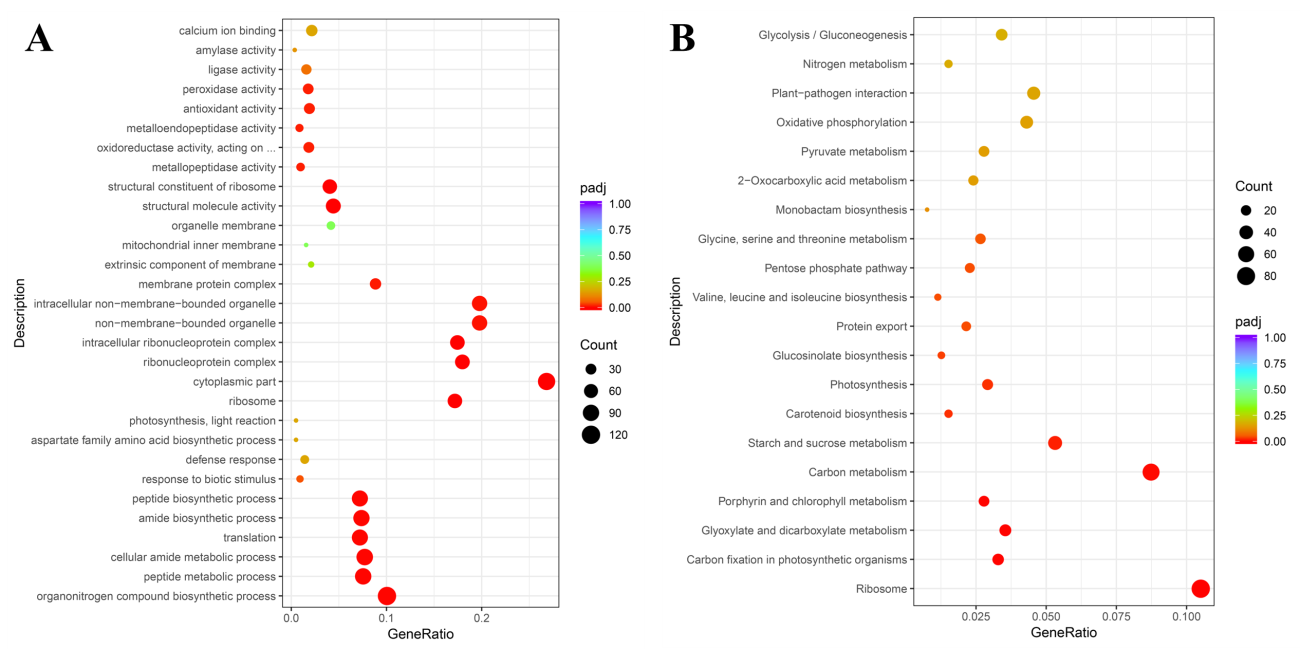
**Figure S9**. GO classifications (A) and KEGG pathways (B) of the DEGs in transgenic plants compared with WT plants under salt stress. X-axis and Y-axis represent the GeneRatio and the term descriptions, respectively. Coloring correlates with the padj. The lower the padj, the more significant the enrichment. Point size correlates with the numbers of DEGs.
